# Reliable Fusion of Stereo Matching and Depth Sensor for High Quality Dense Depth Maps

**DOI:** 10.3390/s150820894

**Published:** 2015-08-21

**Authors:** Jing Liu, Chunpeng Li, Xuefeng Fan, Zhaoqi Wang

**Affiliations:** 1 The Beijing Key Laboratory of Mobile Computing and Pervasive Device, Institute of Computing Technology, Chinese Academy of Sciences, No.6 Kexueyuan South Road Zhongguancun, Haidian District, Beijing 100190, China; E-Mails: cpli@ict.ac.cn (C.L.); fanxuefeng@ict.ac.cn (X.F.); meifeng@ict.ac.cn (Z.W.); 2 University of Chinese Academy of Sciences, No.19A Yuquan Road, Beijing 100049, China

**Keywords:** stereo matching, depth sensor, multiscale pseudo-two-layer model, segmentation, texture constraint, fusion move

## Abstract

Depth estimation is a classical problem in computer vision, which typically relies on either a depth sensor or stereo matching alone. The depth sensor provides real-time estimates in repetitive and textureless regions where stereo matching is not effective. However, stereo matching can obtain more accurate results in rich texture regions and object boundaries where the depth sensor often fails. We fuse stereo matching and the depth sensor using their complementary characteristics to improve the depth estimation. Here, texture information is incorporated as a constraint to restrict the pixel’s scope of potential disparities and to reduce noise in repetitive and textureless regions. Furthermore, a novel pseudo-two-layer model is used to represent the relationship between disparities in different pixels and segments. It is more robust to luminance variation by treating information obtained from a depth sensor as prior knowledge. Segmentation is viewed as a soft constraint to reduce ambiguities caused by under- or over-segmentation. Compared to the average error rate 3.27% of the previous state-of-the-art methods, our method provides an average error rate of 2.61% on the Middlebury datasets, which shows that our method performs almost 20% better than other “fused” algorithms in the aspect of precision.

## 1. Introduction

Depth estimation is one of the most fundamental and challenging problems in computer vision. For decades, it has been important for many advanced applications, such as 3D reconstruction [1], robotic navigation [2], object recognition [3] and free viewpoint television [4]. Approaches for obtaining 3D depth estimation can be distinguished into two categories: passive and active. The goal of passive methods like stereo matching is to estimate a high-resolution dense disparity map by finding corresponding pixels in image sequences [5]. However, these methods heavily rely on how the scene is presented and contain error matchings caused by the luminance variation. Passive methods fail in textureless and repetitive regions where there is not enough visual information to obtain the correspondence. On the contrary, active methods, like depth sensors (ASUS Xtion [6] and Microsoft Kinect [7]), do not suffer from ambiguities in textureless and repetitive regions, because they emit an infrared signal. Unfortunately, sensor errors and the properties of the object surfaces mean that depth maps from a depth sensor are often noisy [8]. Additionally, their resolution is at least an order of magnitude lower than common digital single-lens reflex (DSLR) cameras, which limits many applications. Moreover, they cannot satisfactorily deal with object boundaries and a wide range of distances. Therefore, fusing different kinds of methods using their complementary characteristics undoubtedly makes the obtained depth map more robust and improves the quality. Commonly-used consumer DSLR cameras have higher resolution and can record better texture information than depth sensors. Therefore, it is reasonable to fuse the depth sensor with DSLR cameras to yield a high resolution depth map. Note that for notational clarity, all values mentioned here are disparities (considering that depth values are inversely proportional to disparities).

**Figure 1:**
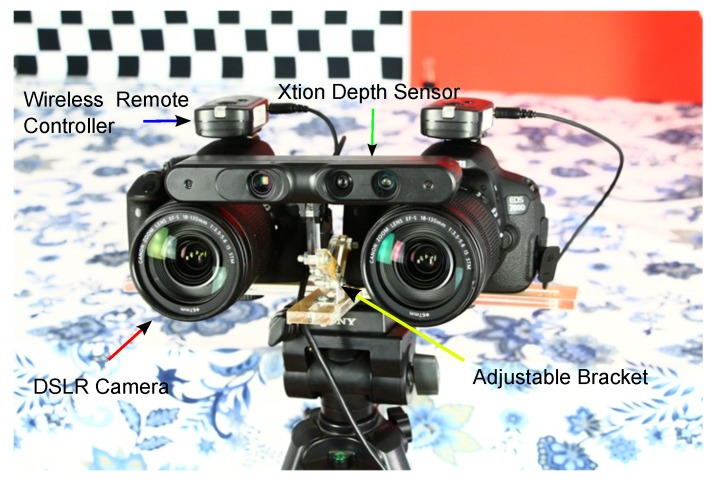
The system used in our method. It consists of two Cannon EOS 700D digital single-lens reflex (DSLR) cameras and one Xtion depth sensor. All DSLR cameras are controlled by the wireless remote controller. We used an adjustable bracket to change the angle and height of the Xtion depth sensor.

**Figure 2:**
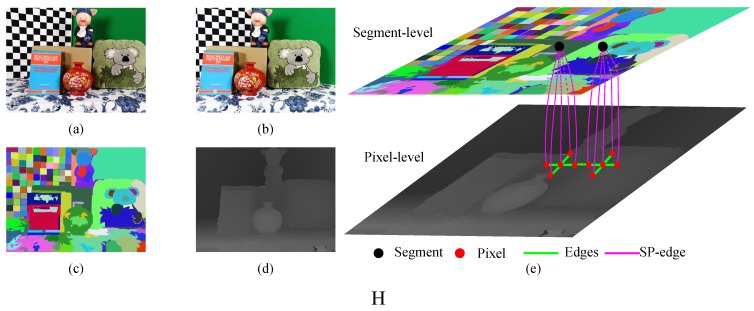
The illustration of the multiscale pseudo-two-layer (MPTL) model. The rectified left (**a**) and right (**b**) images from DLSRs, the segmentation; (**c**) as well as the depth map (**d**) from the depth sensor are taken as our inputs; (**e**) The conceptual structure of our MPTL. The MPTL captures the complementary characteristics of active and passive methods by allowing interactions between them. All interactions are defined to act in the segment-level component, the pixel-level component and the edges that connect them (Segment Pixel-edge, SP-edge). See Section 3.2 for the full details of the MPTL model and Section 4 for further results.

In this paper, we propose a novel disparity estimation method for the system shown in Figure 1. It fuses the complementary characteristics of high resolution DSLR cameras and the Xtion depth sensor to obtain an accurate disparity estimate. Compared to the average error rate 3.27% of the previous state-of-the-art methods, our method provides an average error rate of 2.61% on the Middlebury datasets. It is clear that our method performs almost 20% better than other “fused” algorithms in the aspect of precision. The proposed method views a scene with complex geometric characteristics as a set of segments in the disparity space. It assumes that the disparities of each segment have a compact distribution, which strengthens the smooth variance of the disparities in each segment. Additionally, we assume that each segment is biased towards being a 3D planar surface. The major contributions are as follows:

1. We incorporated texture information as a constraint. The texture variance and gradient is used to restrict the range of the potential disparities for each pixel. In textureless and repetitive regions (which often cause ambiguities when stereo matching), we restrict the possible disparities for a neighborhood centered on each pixel to a limited range around the values suggested by the Xtion. This reduces the errors and strengthens the compact distribution of the disparities in a segment.
2. We propose the multiscale pseudo-two-layer image model (MPTL; Figure 2) to represent the relationships between disparities at different pixels and segments. We consider the disparities from the Xtion as the prior knowledge and use it to increase the robustness to luminance variance and to strengthen the 3D planar surface bias. Furthermore, considering the spatial structures of segments obtained from the depth sensor, we treat the segmentation as a soft constraint to reduce matching ambiguities caused by under- and over-segmentation. Here, pixels with similar colors, but on different objects are grouped into one segment, and pixels with different colors, but on the same object are partitioned into different segments. Additionally, we only retain the disparity discontinuities that align with object boundaries from geometrically-smooth, but strong color gradient regions.

The remainder of this paper is organized as follows. Section 2 gives a summary of various methods used for disparity estimation. We present a pre-processing and some important notations of our model in Section 3.1. We discuss the details of the MPTL image model in Section 3.2, the optimization in Section 3.7 and the post-processing in Section 3.8. Section 4 contains our experiments, and Section 5 presents some conclusions with suggestions for future work.

## 2. Previous Work

There are many approaches to obtaining disparity estimation. They can generally be categorized into two major classes: passive and active. A passive method indirectly obtains the disparity map using image sequences captured by cameras from different viewpoints. Among the plethora of passive methods, stereo matching is probably the most well known and widely applied. Stereo matching algorithms can be divided into two categories [9]: local and global methods. Local methods [10] estimate disparity using color or intensity values in a support window centered on each pixel. However, they often fail around disparity discontinuities and low-texture regions. Global methods [11] use a Markov random field model to formulate the stereo matching as a maximum *a posteriori* probability energy function with explicit smoothness priors. They can significantly minimize matching ambiguities compared to local methods. However, the biggest disadvantage of them is the low computational efficiency. Segmentation-based global approaches [12,13] encode the scene as a set of non-overlapping homogeneous color segments. They are based on the hypothesis that the variance of the disparity in each segment is smooth. In other words, the segment boundaries are forced to coincide with object boundaries. Recently, the ground control point (GCP)-based methods [14] were used as prior knowledge to encode rich information on the spatial structure of the scene. Although a significant number of stereo matching methods have been proposed for obtaining dense disparity estimation, they heavily rely on radiometric variations and assumptions regarding the presentation of the scene. This means that stereo matching often fails in textureless and repetitive regions, where there is not enough visual information to obtain a correspondence. Furthermore, their accuracy is relatively low. Passive methods heavily rely on the luminance condition and how the scene is presented. They often fail in textureless and repetitive regions where there is not enough visual information to obtain the correspondence.

On the contrary, active methods like depth sensors, do not suffer from ambiguities in textureless and repetitive regions, because they emit an infrared signal. Three different kinds of equipments are used in active methods: a laser scanner device, a time-of-flight (ToF) sensor and an infrared single-based device (such as ASUS Xtion [6] and Microsoft Kinect [7]). The laser scanner device [15] can provide extremely accurate and dense depth estimation, but it is too slow to use in real time and too expensive for many applications. The ToF sensor and infrared single-based device can obtain real-time depth estimation and have recently become available from companies, such as 3DV [16] and PMD [17]. However, sensor errors and the properties of the object surfaces mean that depth maps from them are often noisy [8]. Additionally, their resolution is at least an order of magnitude lower than commonly-used DSLR cameras [18], which limits many applications. Moreover, they cannot satisfactorily deal with object boundaries.

It is clear that each disparity acquisition method is limited in some aspects where other approaches may be effective. Joint optimization methods that combine active and passive sensors have been used to make the obtained depth map more robust and to improve the quality. Zhu *et al.* please check throughout [19,20] fused a ToF sensor and stereo cameras to obtain better disparity maps. They improved the quality of the estimated maps for dynamic scenes by extending their fusion technology to the temporal domain. Yang *et al.* [21] presented a fast depth sensing system that combined the complementary properties of passive and active sensors in a synergistic framework. It relied on stereo matching in rich textured regions, while using data from depth sensors in textureless regions. Zhang *et al.* [22] proposed a system that addresses high resolution and high quality depth estimation by fusing stereo matching and a Kinect. A pixel-wise weighted function was used to reflect the reliabilities of the stereo camera and the Kinect. Wang *et al.* [23] presented a novel method that combined the initial stereo matching result and the depth data from a Kinect. Their method also considers the visibilities and pixel-wise noise of the depth data from a Kinect. Gowri *et al.* [24] proposed a global optimization scheme that defines the data and smoothness costs using sensor confidences and the low resolution geometry from a Kinect. They used a spatial search range to limit the scope of the potential disparities at each pixel. The smoothness prior was based on the available low resolution depth data from the Kinect, rather than the image color gradients.

Although existing disparity estimation methods have achieved remarkable results, they are typically performed using pixel-level cues, such as the smoothness of neighboring pixels, and do not consider the regional information (regarding, for example, 3D spatial structure, segmentation and texture) as a cue for the disparity estimation, which is the largest distinction between their method and ours. For example, occlusion cannot be precisely estimated using a single pixel, but a fitted plane-based filling occlusion in a segment can give good results. Additionally, if the spatial structure of neighboring segments is not known, matching ambiguities can arise at the boundaries of neighboring segments that physically belong to the same object, but have different appearances. Without texture information, we cannot be sure if the disparity from stereo matching is more confident than that from the depth sensor in textureless and repetitive regions (where stereo matching usually fails and the depth sensor performs well).

## 3. Method

The proposed method can be partitioned into four phases: pre-processing, problem definition, optimization and post-processing. Each phase will be discussed in detail later.

### 3.1. Pre-Processing

**Figure 3:**
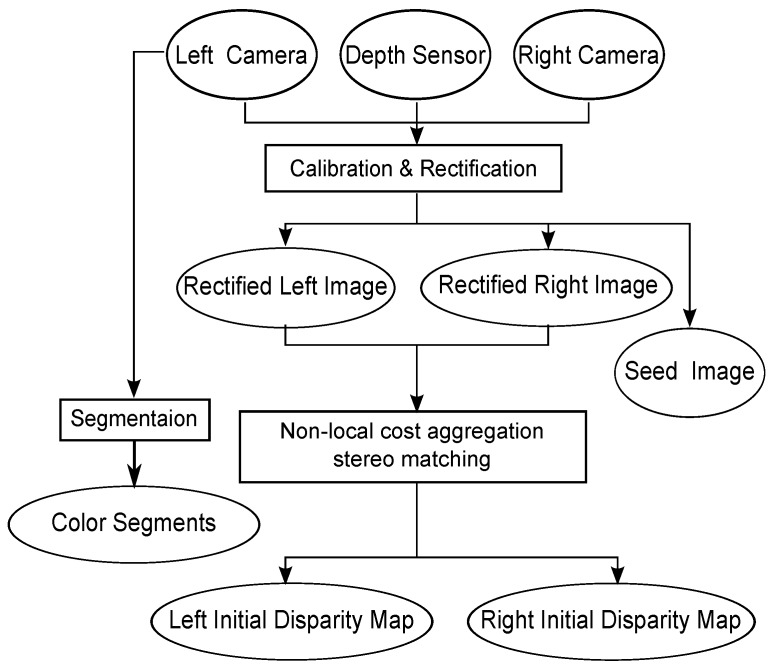
Conceptual flow diagram for the calibration and rectification phase.

There are three camera coordinates involved in our system (Figure 1): the Xtion coordinate, the coordinates of the two DSLR cameras before the epipolar rectification and the DSLR camera coordinates after the epipolar rectification. During the pre-processing step, in order to combine the data from the Xtion and DSLR cameras, as shown in Figure 3, we firstly calibrated two DSLR cameras using the checkerboard-based method [25] and calibrated the DSLR camera pair with the Xtion sensor using the planar surfaces-based method [26], respectively. After the calibration, the depth image obtained from the Xtion is first transformed from the Xtion coordinate to the original DSLR cameras’ coordinates, then rotated and up-sampled, so that it registers with the unrectified left image. Furthermore, according to the theory of epipolar geometric constraints, the registered depth image and original left image, as well as the original right image are rectified to be row-aligned, which means there are only horizontal disparities in the row direction. We denote the seed image (Π) as the map with disparities transferred from the rectified depth map. Each pixel p∈Π is defined as a seed pixel when it is assigned a non-zero disparity. The initial disparity maps ($D_{L}$ and $D_{R}$) of the rectified left and right images ($I_{L}$ and $I_{R}$) are computed using a local stereo matching method [27]. $I_{L}$ is partitioned into a set of segments using the edge-aware filter-based segmentation algorithm [28].

**Figure 4:**
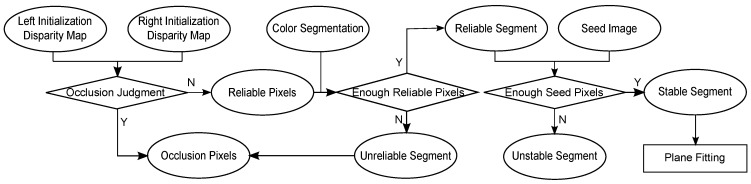
Conceptual flow diagram for the classification phase.

In addition, as shown in Figure 4, all pixels and segments are divided into different categories. The occlusion judgment is used to find the occluded pixels with initial disparity maps ($D_{L}$ and $D_{R}$ of $I_{L}$ and $I_{R}$, respectively) and to classify pixels into different categories: reliable and occluded. As we known, how to find occluded pixels accurately is always the challenging problem, because it often leads to error results that matching points might not even exist at all, especially in depth discontinuities. Pixels are defined as occluded when they are only visible from the left rectified view ($I_{L}$), but not from the right rectified view ($I_{R}$). Since image pairs have been rectified, we assume that occlusion only occurs in the horizontal direction. In early algorithms, cross-consistency checking is often applied to identify occluded pixels by enforcing a one-to-one correspondence between pixels. It is written as:

(1) $$O\left(p\right)=\left\{\begin{array}{cc} 0 & \;|D_{L}\left(p\right)-D_{R}\left(q\right)|<1\\ 1 & \;\mathrm{otherwise} \end{array}\right.\;p\in I_{L},q\in I_{R}$$

$D_{L}\left(p\right)$ and $D_{R}\left(q\right)$ are the disparity of *p* and *q*, and *q* is the corresponding matching point of *p*. If *p* does not meet the cross-consistency checking, then it will be regard as an occluded pixel (O(p)=0); otherwise, *p* is a reliable pixel (O(p)=1). The cross-consistency checking states that a pixel of one image corresponds to at most one pixel of the other image. However, because of different sampling, the projection of a horizontal slant or a curved surface shows various lengths in the image pairs. Therefore, conventional cross-consistency checking that often identifies occluded pixels by enforcing a one-to-one correspondence is only suitable for a frontal parallel surface and cannot be true for a horizontal slant or curved surfaces. Considering the different sampling of image pairs, Bleyer *et al.* [29] proposed a new visibility constraint by extending the asymmetric occlusion model [30] that allows a one-to-many correspondence between pixels. Let $p_{0}$ and $p_{1}$ be neighboring pixels in the same horizontal line of $I_{L}$. Then, $p_{0}$ will be occluded by $p_{1}$ when they meet three conditions:

- $p_{0}$ and $p_{1}$ have the same matching point in $I_{R}$ under their current disparity value;
- $D_{L}\left(p_{0}\right)\leq D_{L}\left(p_{1}\right)$;
- $p_{0}$ and $p_{1}$ belong to different segments.

In this paper, for each pixel *p* of $I_{L}$, if there is only one matching point in $I_{R}$, the conventional cross-checking is applied to obtain the occlusion Equation (1). Otherwise, if there are more than two matching points in $I_{R}$, pixels in $I_{L}$ are marked as either reliable ($O(p_{0})=0$) or occluded ($O(p_{0})=1$), which satisfy or do not satisfy the Bleyer’s asymmetric occlusion model. As shown in Figure 4, each segment belongs to the reliable segment (*R*) if it contains a sufficient amount of reliable pixels; otherwise, it belongs to the unreliable segment ($\bar{R}$). Furthermore, each segment $f_{i}\in R$ is denoted as a stable segment (*S*) when it contains a sufficient number of seed pixels. Otherwise, $f_{i}$ belongs to the unstable segment ($\bar{S}$). We apply a RANSAC-based algorithm to approximate each stable segment $s_{i}\in S$ as a fitted plane $\Psi_{s_{i}}$ using the image coordinates and known disparities of all seed pixels belonging to $s_{i}$. Table 1 lists important notifications used in this paper.

**Table 1:** Notations.

*D*:	Disparity map	D(p):	Disparity value of pixel *p*	Π:	Seed image
$D_{L}$:	Initial disparity map of rectified left image	$D_{R}$:	Initial disparity map of rectified right image	$I_{L}$:	Rectified left DSLR image
$I_{R}$:	Rectified right DSLR image	*R*:	Reliable segment	$\bar{R}$:	Unreliable segment
*S*:	Stable segment	$\bar{S}$:	Unstable segment	$f_{i}$:	*i*-th segment
$s_{i}$:	*i*-th stable segment	$\Psi_{s_{i}}$:	Fitted plane of stable segment $s_{i}$	f(p):	Segment that contains pixel *p*
$f_{i}$:	*i*-th segment	$d_{l}$:	Minimum disparity	$d_{u}$:	maximum disparity
*ϖ*:	Segment boundary pixels	$\Lambda_{l}^{p}$:	Pixel’s potential minimum disparity	$\Lambda_{u}^{p}$:	Pixel’s potential maximum disparity
$\Psi_{i}^{l}$:	Minimum fitted disparity of the *i*-th stable segment	$\Psi_{i}^{u}$:	Minimum fitted disparity of the i-th stable segment		

### 3.2. Problem Formulation

In the problem formulation phase, we propose the MPTL model, which combines the complementary characteristics of stereo matching and the Xtion sensor. As shown in Figure 2, the MPTL model consists of three components:

- The pixel-level component, which improves the robustness against the luminance variance (Section 3.3) and strengthens the smoothness of disparities between neighboring pixels and segments (Section 3.4). Nodes at this level represent reliable pixels from stable and unstable segments. The edges between reliable pixels represent different types of smoothness terms.
- The edge that connects two level components (the SP-edge), which uses the texture variance and gradient as a guide to restrict the scope of potential disparities (Section 3.5).
- The segment-level component, which incorporates the information from the Xtion as prior knowledge to capture the spatial structure of each stable segment and to maintain the relationship between neighboring stable segments (Section 3.6). Each node at this level represents a stable segment.

Existing global methods have achieved remarkable results, but the capability of the traditional Markov random field stereo model remains limited. To lessen the matching ambiguities, additional information is required to formulate an accurate model. In this paper, the pixel-level improved luminance consistency term ($E_{l}$), the pixel-level hybrid smoothness term ($E_{s}$) and the SP-edge texture term ($E_{t}$), as well as the segment-level 3D plane bias term ($E_{p}$) are integrated as additional regularization constraints to obtain a precise disparity estimation (*D*) for a scene with complex geometric characteristics. According to Bayes’ rule, the posterior probability over *D* given *l*, *s*, *t* and *p* is:

(2) $$p\left(D|l,s,t,p\right)=\frac{p(l,s,t,p|D)p(D)}{p(l,s,t,p)}$$

During each optimization process, P(l,s,t,p|D) is only dependent on *l*, *s*, *t* and *p*. Therefore, P(D|l,s,t,p) can be rewritten as:

(3) $$\begin{array}{cc} & p(D|l,s,t,p)\propto p(l|D)p(s|D)p(t|D)p(p|D)p(D) \end{array}$$

Because maximizing this posterior is equivalent to minimizing its negative log likelihood, our goal is to obtain the disparity map (*D*) that minimizes the following energy function:

(4) $$\begin{array}{cc} & E\left(D\right)=E_{l}\left(D\right)+E_{s}\left(D\right)+E_{t}\left(D\right)+E_{p}\left(D\right) \end{array}$$

Each term will be discussed in detail in the following sections.

### 3.3. Improved Luminance Consistency Term

The conventional luminance consistency hypothesis is used to penalize the appearance dissimilarity between corresponding pixels in $I_{L}$ and $I_{R}$, based on the hypothesis that the surface of a 3D object is Lambertian. Because it refers to a perfectly-diffuse appearance in which pixels originating from the same 3D object have similar appearances in different views, its accuracy is heavily dependent on the lighting condition for which colors change substantially depending on the viewpoint. Furthermore, an object may appear to have different colors because different views have different sensor characteristics. In contrast, the Xtion sensor is more robust to the light condition and can be used as prior knowledge to reduce ambiguities caused by the non-Lambertian surface. Thus, the improved luminance consistency term is denoted as:

(5) $$E_{l}=\sum_{p\in I_{L}}\lambda_{l}\cdot \left(1-O\left(p\right)\right)\cdot \left[w_{p}^{l}\cdot C\left(p,q\right)+w_{p}^{x}\cdot X\left(p,q\right)\right]+O\left(p\right)\cdot \lambda_{o}$$

where *q* is the matching pixel of *p* in the other image. O(p) is the asymmetric occlusion function described in Section 3.1, and $\lambda_{o}$ is a positive penalty used to avoid maximizing the number of occluded pixels. C(p,q) is defined as the pixel-wise cost function from stereo matching to measure the color dissimilarity.

(6) $$C\left(p,q\right)=\alpha \cdot \left(1-\exp\left(-\frac{C_{ssd}\left(p,q\right)}{r_{ssd}}\right)\right)+\left(1-\alpha \right)\cdot \left(1-\exp\left(-\frac{C_{g}\left(p,q\right)}{r_{g}}\right)\right)$$

where $r_{ssd}$ and $r_{g}$ are constant values defined by our experience. *α* is the scalar weight from zero to one. $C_{ssd}\left(p,q\right)$ and $C_{g}\left(p,q\right)$ are the color dissimilarity and gradient in three color channels as:

(7) $$C_{ssd}\left(p,q\right)=\sqrt{\sum_{i=R,G,B}{\left(I_{L}^{i}\left(p\right)-I_{R}^{i}\left(q\right)\right)}^{2}}$$

(8) $$C_{g}\left(p,q\right)=\sqrt{\sum_{i=R,G,B}{\left(\nabla I_{L}^{i}\left(p\right)-\nabla I_{R}^{i}\left(q\right)\right)}^{2}}$$

X(p,q) is the components from the Xtion sensor, which are defined as:

(9) $$X\left(p,q\right)=\min\left\{|D\left(p\right)-\Pi \left(p\right)|,T_{\pi }\right\}$$

$T_{\pi }$ is the constant threshold, and D(p) is the disparity value assigned to pixel *p* in each optimization. Π(p) is the disparity of pixel p∈Π. $w_{p}^{x}$ and $w_{p}^{l}$ are pixel-wise confidence weights that are denoted as $w_{p}^{x}=1-w_{p}^{l}$. They are derived from the reliabilities of disparities obtained from stereo matching ($m_{p}^{l}$) and the Xtion ($m_{p}^{x}$) as:

(10) $$w_{p}^{l}=\left\{\begin{array}{cc} \frac{m_{p}^{l}}{m_{p}^{l}+m_{p}^{x}} & \;p\in \Pi \\ 1 & \;otherwise \end{array}\right.$$

where $m_{p}^{l}$ is similar to the attainable maximum likelihood (AML) in [31], which models the cost for each pixel using a Gaussian distribution centered at the minimum actually achieved cost value for that pixel. The reliability of Xtion data $m_{p}^{x}$ is the inverse of the normalized standard deviation of the random error [20]. The confidence of each depth value obtained from the depth sensor decreases with the increasing of the normalized standard deviation.

### 3.4. Hybrid Smoothness Term

The hybrid smoothness term strengthens the segmentation-based assumption that the disparity variance in each segment is smooth and reduces errors caused by under- and over-segmentation. It consists of four terms: the smoothness term for neighboring reliable pixels belonging to the unstable segment ($E_{s_{0}}$), the smoothness term for neighboring reliable pixels in the same stable segment ($E_{s_{1}}$), the smoothness term for neighboring reliable pixels in different stable segments ($E_{s_{2}}$) and the smoothness term for neighboring reliable pixels that belong to stable and unstable segments ($E_{s_{3}}$).

Because there is no prior knowledge about the spatial structure of unstable segments, we define the smoothness term $E_{s_{0}}$ as the conventional second-order smoothness prior Equation (11), which can produce better estimates for a scene with complex geometric characteristics [11].

(11) $$E_{s_{0}}=\sum_{\Phi_{i}^{0}\in \Phi^{0}}\lambda_{s}^{0}\cdot \left[1-exp\left(-\frac{\nabla D(\Phi_{i}^{0})}{\gamma_{s}}\right)\right]\;\left\{p_{0},p_{1},p_{2}\right\}\in \Phi_{i}^{0}$$

where $\gamma_{s}$ and $\lambda_{s}^{0}$ are the geometric proximity and the positive penalty. $\Phi^{0}$ is the set of triple-cliques consisting of consecutive reliable pixels belonging to unstable segment. $\nabla D(\Phi_{i}^{0})$ is the second derivative of the disparity map as:

(12) $$\nabla D\left(\Phi_{i}^{0}\right)=D\left(p_{0}\right)-2D\left(p_{1}\right)+D\left(p_{2}\right).\;\left\{p_{0},p_{1},p_{2}\right\}\in \Phi_{i}^{0}$$

**Figure 5:**
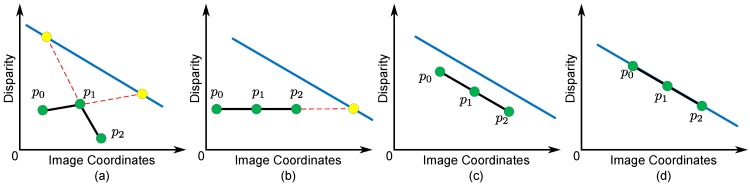
Smoothness term for pixels in the same stable segment ($s_{i}$) with the spatial similarity weight as the triple-clique $\Phi_{i}^{1}=\left\{p_{0},p_{1},p_{2}\right\}$, under different disparity assignments. Given $\Psi_{s_{i}}$ (blue line) as the fitted plane of $s_{i}$. Yellow nodes are the intersect points. (**a**) Case I, D(p0)≠D(p1)≠D(p2) with $\nabla D(\Phi_{i}^{1})\neq 0$; (**b**) Case II, D(p0)=D(p1)=D(p2) with $\nabla D(\Phi_{i}^{1})=0$; (**c**) Case III, D(p0)≠D(p1)≠D(p2) with $\nabla D(\Phi_{i}^{1})=0$; (**d**) Case IV, D(p0)≠D(p1)≠D(p2) with $\nabla D(\Phi_{i}^{1})=0$. As shown in Cases II, III and IV, different disparity assignments correspond to the same second derivative ($\nabla D(\Phi_{i}^{1})=0$).

$E_{s_{0}}$ captures richer features of the local structure and permits planar surfaces without penalty by setting $\nabla D(\Phi_{i}^{0})=0$. However, $E_{s_{0}}$ only considers disparity information when representing the smoothness of neighboring pixels. This means that, in several cases, error matching can result in different disparity assignments, which correspond to the same second derivatives in the disparity map (see Figure 5b–d). Meanwhile, each stable segment can be represented as a fitted plane using the disparity data from the Xtion, which contains prior knowledge about the spatial structure of each stable segment. We can incorporate the spatial similarity weight with the prior knowledge from the Xtion into a conventional second-order smoothness prior. This term encourages constant disparity gradients for pixels in a stable segment and local spatial structures that are similar to the fitted plane of the stable segment. The smoothness term for neighboring reliable pixels in the same stable segment is as follows.

(13) $$E_{s_{1}}=\sum_{\Phi_{i}^{1}\in \Phi^{1}}\lambda_{s}^{1}\cdot \left[2-\delta \left(\Phi_{i}^{1}\right)-\exp\left(-\frac{\nabla D(\Phi_{i}^{1})}{\gamma_{s}}\right)\right]$$

where $\Phi^{1}$ is the set of triple-cliques defined by all 3×1 and 1×3 consecutive reliable pixels along the coordinate direction of the rectified image coordinate in each stable segment. $\lambda_{s}^{1}$ is a positive value penalty. As for the spatial 3D relationship shown in Figure 5, let $s_{i}$ be the stable segment containing $\Phi_{i}^{1}$ and $\Psi_{s_{i}}$ be its corresponding fitted plane. Then, the spatial similarity weight $\delta (\Phi_{i}^{1})$ is denoted as:

(14) $$\delta \left(\Phi_{i}^{1}\right)=\left\{\begin{array}{cc} 0 & \;I:\bar{p_{0}p_{1}}\neq \bar{p_{1}p_{2}}\\ 0.25 & \;\mathrm{II}:\bar{p_{0}p_{1}}=\bar{p_{1}p_{2}}\;\mathrm{and}\;\bar{p_{0}p_{2}}\cap \Psi_{s_{i}}\\ 0.5 & \;\mathrm{III}:\bar{p_{0}p_{1}}=\bar{p_{1}p_{2}}\;\mathrm{and}\;\bar{p_{0}p_{2}}//\Psi_{s_{i}}\\ 1 & \;\mathrm{IV}:\bar{p_{0}p_{1}}=\bar{p_{1}p_{2}}\;\mathrm{and}\;\bar{p_{0}p_{2}}\in \Psi_{s_{i}} \end{array}\right.\;\left\{p_{0},p_{1},p_{2}\right\}\in \Phi_{i}^{1}$$

- Case I: When $\bar{p_{0}p_{1}}\neq \bar{p_{1}p_{2}}$, the disparity gradients of pixels in $\Phi_{i}^{1}$ are not constant ($\nabla D(\Phi_{i}^{1})\neq 0$). This case violates the basic segmentation assumptions that the disparity variance of neighboring pixels is smooth, so a large penalty is added to prevent it from happening in our model (see Figure 5a).
- Cases II, III and IV: When $\bar{p_{0}p_{1}}=\bar{p_{1}p_{2}}$, the disparity gradients of pixels in $\Phi_{i}^{1}$ are constant ($\nabla D(\Phi_{i}^{1})=0$). This means that the variance of the disparities is smooth. Furthermore, our model checks the relationship between all pixels in $\Phi_{i}^{1}$ and $\Psi_{s_{i}}$ (see Figure 5b–d). $\delta (\Phi_{i}^{1})$ does not penalize the disparity assignment if all pixels in $\Phi_{i}^{1}$ belong to $\Psi_{s_{i}}$ (Case IV in Figure 5d), because it is reasonable to assume that the local structure of $\Phi_{i}^{1}$ is the same as the spatial structure of $\Psi_{s_{i}}$. Note that we impose a larger penalty to Case II than to Case III to strengthen the similarity between the spatial structure of $\Phi_{i}^{1}$ and $\Psi_{s_{i}}$.

In some segmentation-based algorithms [32], the segmentation is implemented as a hard constraint by setting $\lambda_{s}^{0}$ and $\lambda_{s}^{1}$ to be positive infinity. This does not allow any large disparity variance within a segment. In other words, each segment can only be represented as a single plane model, and the boundaries of a 3D object must be exactly aligned with segment boundaries. Unfortunately, not all segments can be accurately represented as a fitted plane, and not all 3D object boundaries coincide with segment boundaries. The accuracy of the segmentation-based algorithms is easily affected by the initial segmentation. On the one hand, the initial segmentation typically contains some under-segmented regions (where pixels from different objects, but with similar colors are grouped into one segment). As a direct consequence of under-segmentation, foreground and background boundaries are blended if they have similar colors at disparity discontinuities. To avoid this, we use segmentation as a soft constraint by setting $\lambda_{s}^{0}$ and $\lambda_{s}^{1}$ to be positive finite, so that each segment can contain arbitrary fitted planes.

On the other hand, pixels with different colors, but on the same object are over-segmented into different segments in the initial segmentation, which causes computationally inefficiency and ambiguities on segment boundaries. In this paper, we considered the spatial structure of neighboring stable segments using disparities from the Xtion. Therefore, we apply the smoothness term for neighboring pixels belonging to different stable segments ($E_{s_{2}}$) to avoid errors caused by the over-segmentation. Let *p* and *q* be neighboring pixels belonging to stable segments $s_{i}$ and $s_{j}$, respectively, Then, $E_{s_{2}}$ can be expressed as:

(15) $$E_{s_{2}}=\left\{\begin{array}{cc} 0 & \;I:\Psi_{s_{i}}\neq \Psi_{s_{j}}\;\mathrm{and}\;D\left(p\right)\neq D\left(q\right)\\ \lambda_{s}^{2} & \;\mathrm{II}::\Psi_{s_{i}}\neq \Psi_{s_{j}}\;\mathrm{and}\;D\left(p\right)=D\left(q\right)\\ \lambda_{s}^{2} & \;\mathrm{III}::\Psi_{s_{i}}=\Psi_{s_{j}}\;\mathrm{and}\;D\left(p\right)\neq D\left(q\right)\\ 0 & \;\mathrm{IV}:\Psi_{s_{i}}=\Psi_{s_{j}}\;\mathrm{and}\;D\left(p\right)=D\left(q\right) \end{array}\right.$$

As shown in Equation (15), for Cases I and II, if $\Psi_{s_{i}}$ is not equal to $\Psi_{s_{j}}$, this means that $s_{i}$ and $s_{j}$ have different spatial structures, and the 3D object boundary coincides with the boundary between them. The disparity variance between *p* and *q* is allowed without any penalty (Case I); otherwise, a constant penalty $\lambda_{s}^{2}$ is added (Case II). In contrast, for Cases III and IV, if $\Psi_{s_{i}}$ is equal to $\Psi_{s_{j}}$, this means that $s_{i}$ and $s_{j}$ have different appearances, but have similar spatial structures and belong to the same 3D object. In these two cases, the disparity variance between *p* and *q* is not allowed by adding a penalty. $E_{s_{2}}$ reduces the ambiguities caused by over-segmentation and retains only the disparity discontinuities that are aligned with object boundaries from geometrically-smooth, but strong color gradient regions, where pixels with different colors, but from the same object are partitioned into different segments.

Because unstable segments do not have sufficient disparity information from the Xtion to regard their spatial plane models, the smoothness term for neighboring pixels that belong to the stable and unstable segments ($E_{s_{3}}$) encourages neighboring pixels to take the same disparity assignment. It takes the form of a standard Potts model,

(16) $$E_{s_{3}}=\left\{\begin{array}{cc} 0 & \;D(p)=D(q)\\ \lambda_{s}^{3} & \;D(p)\neq D(q) \end{array}\right.\;p\in S,q\in \bar{S}$$

Thus, let *ϖ* be the set of pixels belonging to segment boundaries, the hybrid smoothness term is:

(17) $$E_{s}=\left\{\begin{array}{cc} E_{s_{0}} & \left\{p_{0},p_{1},p_{2}\right\}\in \Phi_{i}\mathrm{and}\Phi_{i}\in \bar{S}\mathrm{and}\Phi_{i}\cap \varpi =\emptyset \\ E_{s_{1}} & \left\{p_{0},p_{1},p_{2}\right\}\in \Phi_{i}\mathrm{and}\Phi_{i}\in S\mathrm{and}\Phi_{i}\cap \varpi =\emptyset \\ E_{s_{2}} & \left\{p_{0},p_{1},p_{2}\right\}\in \Phi_{i}\mathrm{and}\Phi_{i}\cap \varpi \neq \emptyset \mathrm{and}\left\{p_{0},p_{1},p_{2}\right\}\in S\\ E_{s_{3}} & \left\{p_{0},p_{1},p_{2}\right\}\in \Phi_{i}\mathrm{and}\Phi_{i}\cap \varpi \neq \emptyset \mathrm{and}\left\{p_{0},p_{1}\right\}\in S,\left\{p_{2}\right\}\in \bar{S} \end{array}\right.$$

### 3.5. Texture Term

Stereo matching often fails in textureless and repetitive regions, because there is not enough visual information to obtain a correspondence. However, the Xtion does not suffer from ambiguities in these regions. Therefore, the disparities from the Xtion are more reliable than those obtained from stereo matching on textureless and repetitive regions and should be closer to the range of potential disparities for pixels in these regions. In contrast, the disparities from the Xtion are susceptible to noise and problems caused by rich texture regions and have poor performance in preserving object boundaries. Therefore, the disparities obtained from stereo matching are more reliable than that of the Xtion and should be used to define the scope of potential disparities of pixels in those regions. Considering the complementary characteristics of stereo matching and the Xtion sensor, texture information can be used as a useful guide for disparities.

**Figure 6:**
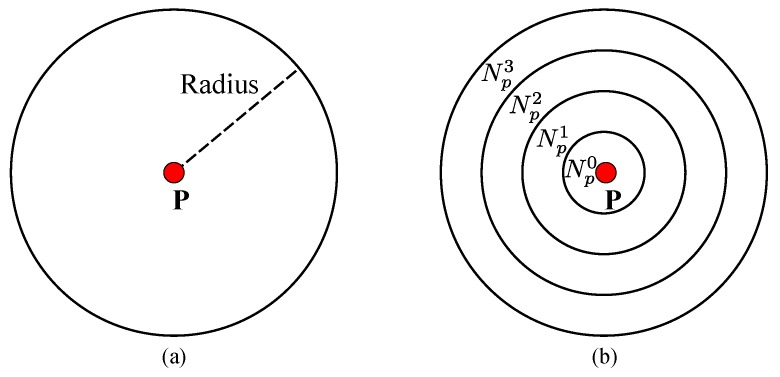
Surrounding neighborhood patch, $N_{p}$, for: (**a**) pixel p and (**b**) its corresponding sub-regions.

The texture variance and gradient are used as a cue to restrict the scope of potential disparities for pixels. This reduces errors caused by noise or outliers and makes the distribution of the disparity more compact. To do this, we first define a surrounding neighborhood patch $N_{p}$ (with a radius of, for example, 20 pixels) centered at each pixel $p\in I_{L}$, as shown in Figure 6. Considering that the annular spatial histogram is translation and rotation invariant [33], $N_{p}$ is evenly partitioned into four annular sub-regions. For each sub-region $N_{p}^{i}\left(i=0\cdots 3\right)$, we compute its normalized intensity 16-bin gray histogram $H_{p}^{i}=\left\{h_{p}^{\left(i,j\right)},j=0\cdots 15\right\}$ to represent the annular distribution density of $N_{p}$ as a 64-dimensional feature vector.

Finally, let $L_{p}$ be a 1D line segment ranging from (p−10) to (p+10) in the same row of *p* in $I_{L}$. The texture variance and gradient of *p* is determined by the texture dissimilarity $\Gamma_{p}$ Equation (18), using the Hamming distance Equation (19) between the annular distribution densities of *p* and its neighboring pixel *q* in $L_{p}$. That is,

(18) $$\Gamma_{p}=min_{\left(q\in L_{p},q\neq p\right)}\sum_{i=0}^{3}\sum_{j=0}^{15}H\left(h_{p}^{\left(i,j\right)},h_{q}^{\left(i,j\right)}\right)$$

(19) $$H\left(h_{p}^{\left(i,j\right)},h_{q}^{\left(i,j\right)}\right)=\left\{\begin{array}{c} 1\;|h_{p}^{\left(i,j\right)}-h_{q}^{\left(i,j\right)}|\geq T_{H}\\ 0\;\;\mathrm{otherwise} \end{array}\right.$$

Each pixel’s disparity variance buffer ($\Omega_{p}$) can be denoted as:

(20) $$\Omega_{p}=1+\left[1-exp\left(-\frac{\Gamma_{p}}{\gamma_{H}}\right)\right]\cdot \xi \;\xi =0.2\cdot \left(d_{u}-d_{l}\right)$$

$d_{l}$ and $d_{u}$ are the minimum and maximum disparities. $\Gamma_{p}$ is small in the textureless and repetitive regions and is large in the rich texture regions or object boundaries. The scope of each pixel’s potential disparities [$\Lambda_{l}^{p}$, $\Lambda_{u}^{p}$] is denoted as:

(21) $$\Upsilon_{l}=\max\left\{\left(\Psi_{f(p)}^{l}-\Omega_{p}\right),d_{l})\right\}$$

(22) $$\Upsilon_{u}=\min\left\{\left(\Psi_{f(p)}^{l}+\Omega_{p}\right),d_{u})\right\}$$

(23) $$\begin{array}{cc} & \Lambda_{l}^{p}=\left\{\begin{array}{cc} \max\left\{\left(\Theta_{l}^{p}-\Omega_{p}\right),\Upsilon_{l}\right\} & \chi_{p}\geq T_{\Lambda }\;\mathrm{and}\;f\left(p\right)\in S\\ \Upsilon_{l} & \chi_{p}<T_{\Lambda }\;\mathrm{and}\;f\left(p\right)\in S\\ d_{l} & f\left(p\right)\in \bar{S} \end{array}\right. \end{array}$$

(24) $$\begin{array}{cc} & \Lambda_{u}^{p}=\left\{\begin{array}{cc} \min\left\{\left(\Theta_{u}^{p}+\Omega_{p}\right),\Upsilon_{u}\right\} & \chi_{p}\geq T_{\Lambda }\;\mathrm{and}\;f\left(p\right)\in S\\ \Upsilon_{u} & \chi_{p}<T_{\Lambda }\;\mathrm{and}\;f\left(p\right)\in S\\ d_{u} & f\left(p\right)\in \bar{S} \end{array}\right. \end{array}$$

where $\Theta_{l}^{p}$ and $\Theta_{u}^{p}$ are the minimum and maximum disparities from the Xtion in the region centered at *p* in $I_{L}$. f(p) is the segment that contains *p*. $\Psi_{f(p)}^{l}$ and $\Psi_{f(p)}^{u}$ are the minimum and maximum fitted disparities of f(p). $\chi_{p}$ is the number of seed pixels in the region centered at *p*. $T_{\Lambda }$ is a positive value. As described in Equation (23), there are three cases for the definition of $\Lambda_{l}^{p}$:

- When f(p) is a stable segment (f(p)∈S) and contains sufficient seed pixels ($\chi_{p}>T_{\Lambda }$), $\Lambda_{l}^{p}$ is equal to $\max\left\{\left(\Theta_{l}^{p}-\Omega_{p}\right),\Upsilon_{l}\right\}$. In this case, there are enough seed pixels from the Xtion to denote a guide for the variance of disparities of *p*. If *p* is in the textureless or repetitive region, $\Omega_{p}$ is small. This indicates that stereo matching may fail in these regions, and a small search range should be used around disparities from the Xtion. In contrast, if *p* is in the rich textured region or object boundaries, $\Omega_{p}$ is large. This indicates that disparities from the Xtion may be susceptible to noise and problems caused by rich texture regions where disparities obtained from stereo matching are more reliable. Then, a broader search range should be used, so that we can extract better results not observed by the Xtion.
- When f(p) is a stable segment (f(p)∈S), but there are not enough seed pixels around *p* ($\chi_{p}\leq T_{\Lambda }$), $\Lambda_{l}^{p}$ is equal to $\Upsilon_{l}$. In this case, although there are some seed pixels from the Xtion, they are not enough to represent the disparity variance around *p*. On the other hand, because each stable segment is viewed as a 3D fitted plane, the search range for the potential disparities is limited by the fitted disparity of f(p) and the disparity variance buffer ($\Omega_{p}$).
- When f(p) is an unstable segment ($f\left(p\right)\in \bar{S}$), $\Lambda_{l}^{p}$ is the minimum disparity ($d_{l}$).

Similarly, $\Lambda_{u}^{p}$ can be obtained in the same way. Then, the SP-edge term (which defines the scope of pixel’s potential disparities) is:

(25) $$E_{t}=\left\{\begin{array}{c} 0\;\Lambda_{l}^{p}\leq D\left(p\right)\leq \Lambda_{u}^{p}\\ \lambda_{t}\;\mathrm{otherwise} \end{array}\right.$$

### 3.6. 3D Plane Bias Term

This 3D plane bias term focuses on strengthening the assumption that each stable segment has a 3D plane bias. It is denoted as:

(26) $$E_{p}=\sum_{s_{i}\in S}\sum_{p\in s_{i}}\lambda_{p}\cdot \min\left\{|D\left(p\right)-\Psi_{s_{i}}\left(p\right)|,T_{p}\right\}$$

where D(p) is the assigned value of pixel *p* in $I_{L}$. $\Psi_{s_{i}}\left(p\right)$ is the plane fitted value, and $T_{p}$ is a threshold value. Note that for notation clarity, the traditional 3D bias assumption is a hard constraint that forbids any distinctive between D(p) and $\Psi_{s_{i}}\left(p\right)$ by setting $\lambda_{p}$ to be infinite. On the contrary, our 3D plane bias term is a soft constraint that a certain distinctive between D(p) and $\Psi_{s_{i}}\left(p\right)$ is allowed by setting $\lambda_{p}$ to be a finite positive value.

### 3.7. Optimization

The energy function defined in Equation (4) is a function of the real discrete disparity map. In this section, we describe how to optimize Equation (4) using the fusion move algorithm to obtain the disparity map $D^{*}$:

(27) $$D^{*}=argmin_{D}E\left(D\right)$$

The fusion move approach [34] is an extended approach of the α−expansion algorithm [35], which allows arbitrary values for each pixel in the proposed disparity map. It generates a new result by fusing the current and proposed disparity maps with the energy either decreasing or remaining constant. Let $D^{c}$ and $D^{p}$ be the current and proposed disparity maps of $I_{L}$. Our goal is to optimally “fuse” $D^{c}$ and $D^{p}$ to generate a new depth map $D^{n}$, so that the energy $E(D^{n})$ is lower than $E(D^{c})$. This fusion move is achieved by taking each pixel in $D^{n}$ from either $D^{c}$ or $D^{p}$, according to a binary indicator map *B*. *B* is the result of the graph cut-based fusion move Markov random field optimization technique. During each optimization, each pixel either keeps its current disparity value (B(p)=0) or changes it to proposed disparity value (B(p)=1). That is,

(28) $$D^{n}=\left(1-B\right)\cdot D^{c}+B\cdot D^{p}$$

However, the fusion move is limited to optimizing the submodular binary fusion-energy functions that consist of unary and pairwise potentials. Because of the hybrid smoothness term, our binary fusion-energy functions are not submodular and cannot be directly solved using the fusion move [36]. Using the quadratic pseudo-Boolean optimization (QPBO) algorithm [37], we can obtain a partial solution for the non-submodular binary fusion-energy function by assigning either zero or one to partial pixels, and leaving the rest unassigned. The partial solution is a part of the global minimum solution, and its energy is not higher than that of the original solution. Because of the given lowest average number of unlabeled pixels, we used Quadratic Pseudo Boolean Optimization with Probing (QPBO-P) [38] and Quadratic Pseudo Boolean Optimization with Improving (QPBO-I) [39] as our fusion strategies. During the optimization, the pixel-level improved luminance consistency term ($E_{l}$), the SP-edge texture term ($E_{t}$) and the segment-level 3D plane bias term ($E_{p}$) are expressed as unary terms, respectively. We tackle the transformation problem of the pixel-level hybrid smoothness term ($E_{s}$) that contains triple-cliques using the decomposition method called Excludable Local Configuration (ELC) [40]. The essence of the ELC method is a QPBO-based transformation of a general higher-order Markov random field with binary labels into a first-order one that has the same minima as the original. It combines a new reduction with the fusion move and QPBO to approximately minimize higher-order multi-label energies. Furthermore, the new reduction technique is along the lines of the Kolmogorov-Zabih reduction that can reduce any higher-order minimization problem of Markov random fields with binary labels into an equivalent first-order problem. Each triple clique in $E_{s}$ is decomposed into a set of unary or pairwise terms by ELC without introducing any new variables.

The choice of the proposed disparity maps in the fusion move approach is another crucial factor for the successful use and efficiency of the fusion move. Because there is not an algorithm that can be applied to all situations, our goal is to expect all proposed disparity maps to be correct in some parts and under some parameter setting. Here, we use the following schemes to obtain all proposed disparity maps:

- Proposal A: Uniform value-based proposal. All disparities in the proposal are assigned to a discrete disparity, in the range of $d_{l}$ to $d_{u}$.
- Proposal B: The hierarchical belief propagation-based algorithm [41] is applied to generate proposals with different segmentation maps.
- Proposal C: The joint disparity map and color consistency estimation method [42], which combines mutual information, a SIFT descriptor and segment-based plane-fitting techniques.

During each optimization, the result of the current fusion move is used as the initial disparity map of the next iteration.

### 3.8. Post-Processing

The post-processing is composed of two steps: filling occlusions and refinement. Given that *p* is a occluded pixel in $I_{L}$, a two-step method is implemented to estimate disparities of occluded pixels. If f(p)∈S, the fitted plane value $\Psi_{f(p)}\left(p\right)$ is assigned as *p*’s disparity. Otherwise, the disparity of *p* is the smaller disparity of its closet left and right seed pixels that belongs to the background.

After filling occlusions, in order to obtain an accurate disparity map and to remove ambiguities at object boundaries, the weighted joint bilateral filter with the slope depth compensation filter [43] is applied to refine the disparity map.

## 4. Results and Discussion

Here, a series of evaluations were performed to verify the effectiveness and accuracy of the proposed method. Results were composed of qualitative and quantitative analyses. The segmentation parameters for all experiment are the same: spatial bandwidth =7, color bandwidth =6.5, minimum region =20. Other parameters are presented in Table 2. They were kept constant for all experiments and were typically empirically based.

**Table 2:** Parameter settings for all experiments.

$T_{H}$	$T_{\Lambda }$	$T_{\pi }$	$T_{p}$	$\gamma_{H}$	$\gamma_{s}$	$\lambda_{t}$	$\lambda_{l}$	$\lambda_{o}$	$\lambda_{s}^{0}$	$\lambda_{s}^{1}$	$\lambda_{s}^{2}$	$\lambda_{s}^{3}$	$\lambda_{p}$
0.1	50	3	5	35	1.5	200	12	200	40	40	15	15	10

### 4.1. Qualitative Evaluation Using the Real-World Datasets

We performed qualitative analyses of the proposed method using real-world datasets. In all evaluations, we captured the image pairs using the system in Figure 1 and regarded the left DSLR cameras as the target to be estimated by the disparity map. Notice that all scenes contain weakly-textured and repetitive regions, as well as a non-Lambertian surface.

**Figure 7:**
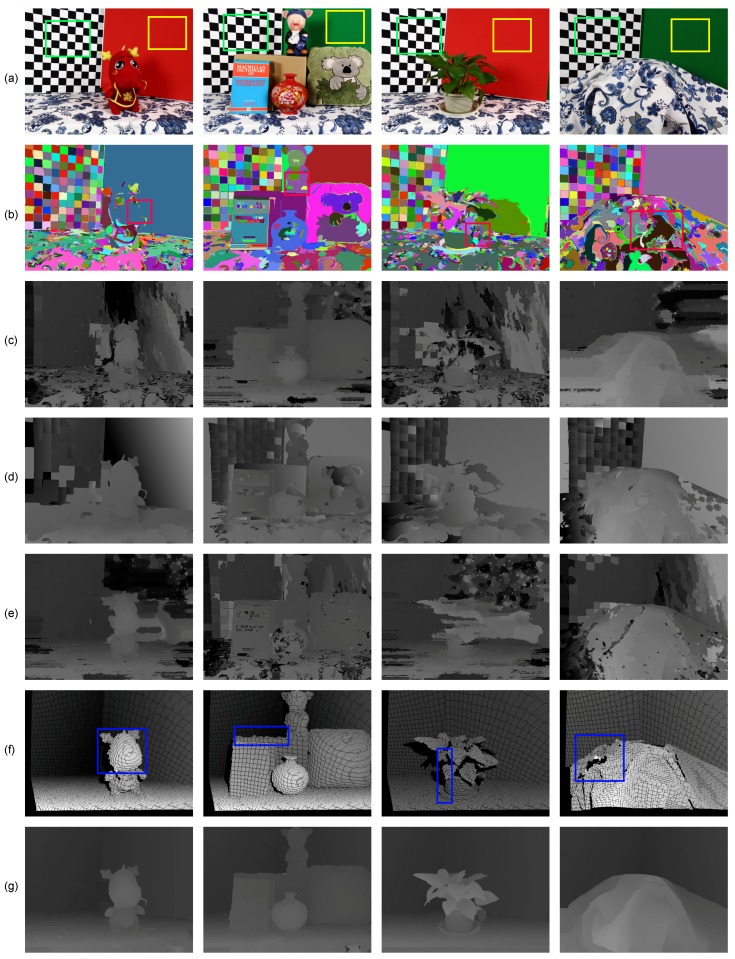
Results of the different methods applied to the real-world scenes: Dragon, Book, Plant and Tablecloth. Each column from up to down is: (**a**) the rectified left image; (**b**) the segmentation result, (**c**) the disparity map of FCVF [44]; (**d**) the disparity map of SOSP [11]; (**e**) the disparity map of segment tree (ST) [13]; (**f**) the seed image transformed from the Xtion data and (**g**) our result.

**Figure 8:**
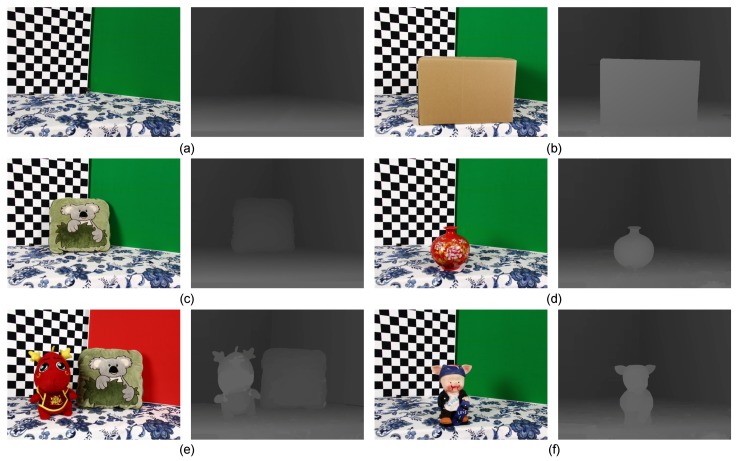
Comparative results for different real-world scenes: (**a**) Board; (**b**) Box; (**c**) Kola; (**d**) Vase; (**e**) Dragon and Kola; (**f**) Piggy. All scenes were approximately 0.5–1.5 m from the cameras, and the maximum disparity was 107 pixels. Each scene from left to right contains the rectified left image and its associated result of our method.

In order to illustrate that our method combines the complementary characteristics of the various disparity estimation methods and outperforms using the conventional stereo matching or depth sensor alone, we evaluated the qualitative quality of the disparity estimates from three stereo matching methods, the Xtion depth sensor and the proposed method using several complex indoor scenes in Figure 7. As show in Figure 7c, although the local stereo matching with fast cost volume filtering (FCVF [44]) performed well by recovering the object boundaries using a color image as a guide, it was very fragile for noise and textureless regions (such as the uniformly-colored board in the yellow rectangle of Figure 7a). In contrast, the depth values obtained from the active sensor are more accurate (see the same regions in Figure 7f). Therefore, our method overcame these problems with the improved luminance consistency term and texture term by incorporating the prior depth information from the depth sensor. As shown in Figure 7d, segmentation-based global stereo matching with second order smoothness prior (SOSP [11]) overcame some of the problems caused by the noise and outliers, but did not solve the problems caused by the over-segmentation, which led to ambiguous matching when segment boundaries did not correspond to object boundaries (green rectangle in Figure 7a). Segment tree-based stereo matching (ST [13]) blended the foreground and background in the under-segmented region (as shown in Figure 7e), where different objects with similar appearances (such as the red rectangle in Figure 7b) were grouped into a segment. Comparing to our result and those of segmentation-based methods, it is clear that the proposed hybrid smoothness term helps reduce matching ambiguities causing by over-segmentation and under-segmentation with the indication of the depth sensor. The raw data from the depth sensor were noisy and had poor performance in preserving object boundaries (blue rectangle in Figure 7f); our result is more robust in this situation and can be used to improve the performance of the depth sensor by considering the color and segmentation information from stereo matching. Based on the above, we can safely draw the conclusion that the proposed method obtains accurate depth estimation by combining the complementary characteristics of stereo matching and the depth sensor. We also tested the proposed method on other real-world scenes to verify its robustness (see Figure 8).

**Figure 9:**
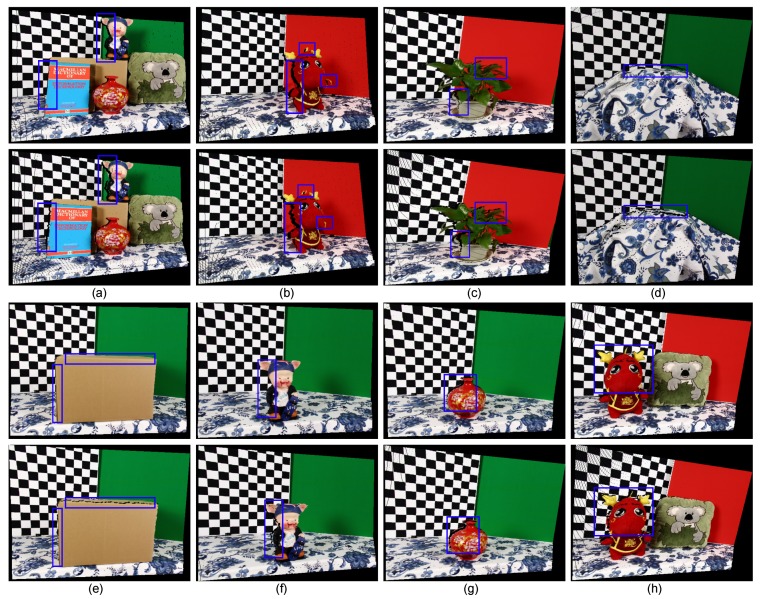
Comparative results for 3D reconstructions. (**a**) Book; (**b**) Dragon; (**c**) Plant; (**d**) Tablecloth; (**e**) Box; (**f**) Piggy; (**g**) Vase; (**h**) Dragon and Kola. Each scene from top to bottom contains the reconstruction using our method and the result using the up-sampled disparity map captured by the Xtion depth sensor.

Furthermore, we implemented the post-processing processing introduced in Section 3.8 to assign valid disparities to pixels in the black regions of seed images. The seed image after assignment can be treated as the up-sampling disparity map of the target image captured by the Xtion alone. Then, we evaluated the quality of the 3D reconstruction from our method and that using only Xtion data (see Figure 9). The 3D point cloud reconstructions consist of the pixels’ image coordinates and their associated disparities in disparity space. The blue rectangles highlight some regions where our method performed well. For example, the proposed method was more effective at retaining the boundaries of Piggy and Plant (Figure 9a,c) and correctly recovered the top of the head and beard of the dragon (Figure 9b). These comparisons illustrate that the stereo matching using the the depth sensor as the prior knowledge is more effective and accurate than using stereo matching or the depth sensor alone.

### 4.2. Quantitative Evaluation Using the Middlebury Datasets

**Figure 10:**
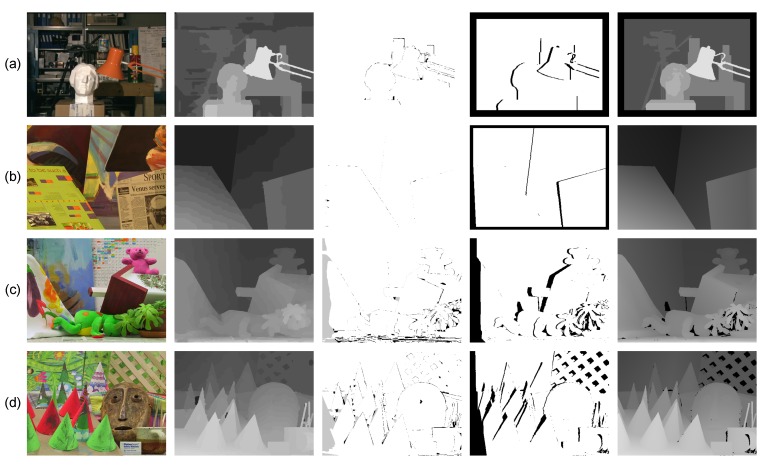
Evaluation results on the Middlebury standard data. (**a**) Tsukuba; (**b**) Venus; (**c**) Teddy; (**d**) Cones. Each row contains (from left to right): the left image, our results, the error map (error matching pixels whose absolute disparity errors are larger than one in non-occlusion and occlusion regions are marked in black and gray), the occlusion map (occluded pixels are marked black) and the ground truth map.

**Figure 11:**
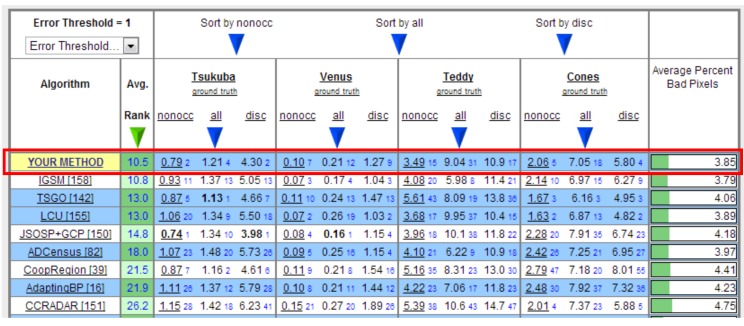
Middlebury results of our method. All numbers are the percentage of error pixels whose absolute disparity error is larger than one. The blue number is the ranking in every column. Our method outperforms the conventional stereo matching algorithms and ranks first among approximately 164 methods according to the average of the sum of the rankings in every column (up to 20 April 2015).

To quantitatively illustrate the validity of the proposed method, we also conducted evaluations on the Middlebury datasets [9,45] and focused on recovering the disparity map of the left image in each dataset. The evaluation is made by third-size resolution Views 1 and 5 of all image pairs. However, because there is nothing about the scanning depth information of this dataset, we used the method described in [14] to simulate the seed image transformed from the Xtion projected to View 1. This technique is based on a voting strategy and simply requires some disparity maps produced using several stereo methods [46,47,48]. Each pixel was labeled as a seed if its disparity in different maps was consistent (varied by less than a fixed threshold and was not near the intensity edge). Results on these datasets and their corresponding errors (compared with the ground truth) in non-occlusion regions are shown in Figure 10. As shown in Figure 11, our method ranks first among approximately 164 methods listed on the website [49]. It performs especially well on the Tsukuba image pairs, with minimum errors in non-occluded regions and near depth discontinuities.

**Figure 12:**
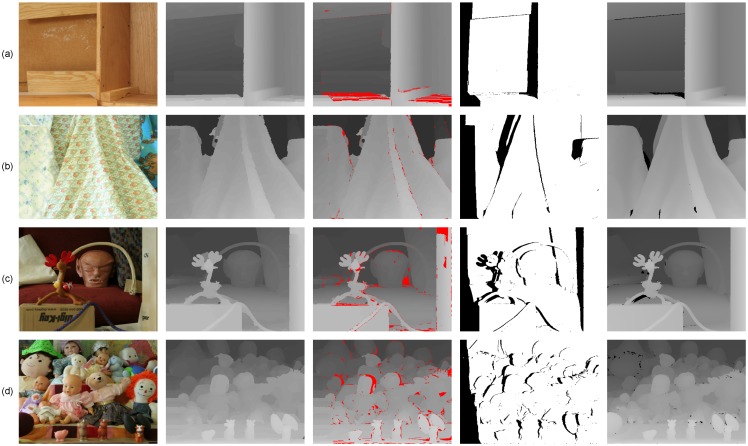
Evaluation results on the Middlebury extension datasets. (**a**) Wood1; (**b**) Cloth4; (**c**) Reindeer; (**d**) Dolls. Each row contains (left to right): the left image, our results, the error map (from top to bottom, the percentages of error pixels with absolute disparity error larger than one in non-occlusion regions are: 4.35%, 1.03%, 4.31%, 4.78%; error pixels are marked red), the occlusion map (occluded pixels are marked black) and the ground truth map.

**Figure 13:**
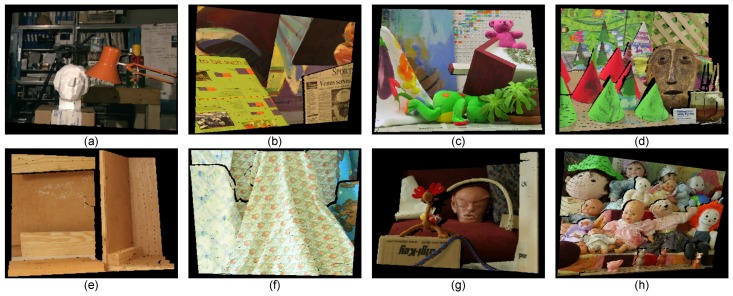
The results of 3D reconstructions. (**a**) Tsukuba; (**b**) Venus; (**c**) Teddy; (**d**) Cones; (**e**) Wood1; (**f**) Cloth4; (**g**) Reindeer; (**h**) Dolls.

On the other hand, as shown in Figure 12, we presented some evaluation results of the Middlebury extension datasets [50,51] to illustrate the robustness of the proposed method. Meanwhile, we also show the quality of 3D reconstruction of Middlebury datasets using the pixels’ image coordinate and their corresponding disparities in disparity space (see Figure 13). The evaluation results in Figure 12 and Figure 13 illustrate that our method is robust to different types of scenes and outperforms in slanted and highly curved surfaces.

**Figure 14:**
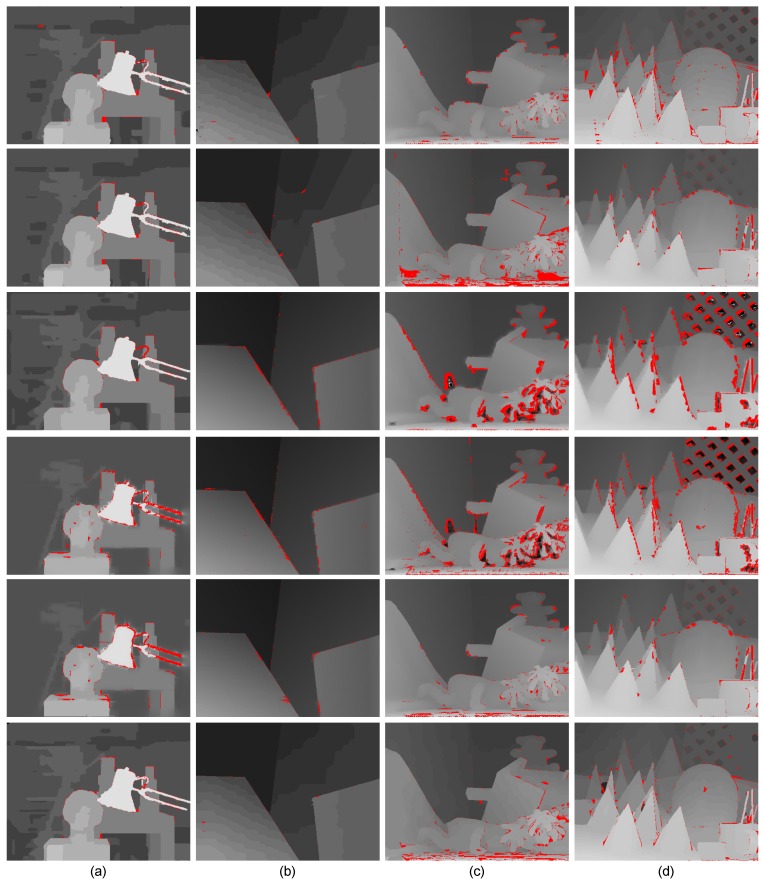
Evaluation results with the state-of-the-art “fused” scheme-based algorithms on the Middlebury datasets. (**a**) Tsukuba; (**b**) Venus; (**c**) Teddy; (**d**) Cones. Each column from top to bottom is the results obtained from: Zhu *et al.* [20], Wang *et al.* [23], Yang *et al.* [52], Jaesik *et al.* [53,55], James *et al.* [54] and our method. Error pixels with absolute disparity error larger than one in non-occlusion regions are marked red. The percentages of error pixels are listed in Table 3.

**Figure 15:**
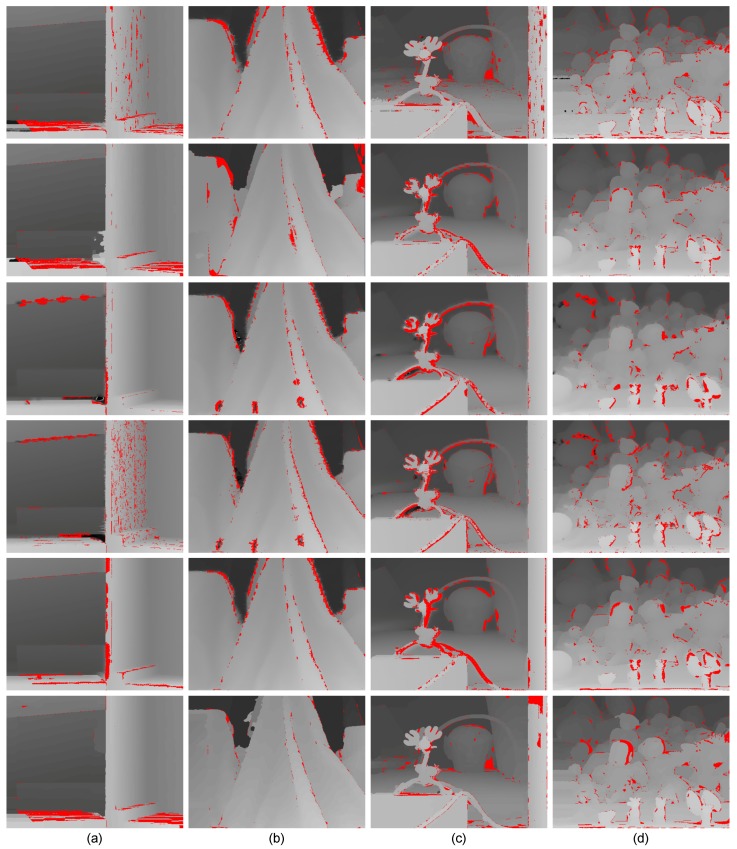
Evaluation results with the state-of-the-art “fused” scheme-based algorithms on the Middlebury extension datasets. (**a**) Wood1; (**b**) Cloth4; (**c**) Reindeer; (**d**) Dolls. Each column from top to bottom is the results obtained from: Zhu *et al.* [20], Wang *et al.* [23], Yang *et al.* [52], Jaesik *et al.* [53,55], James *et al.* [54] and our method. Error pixels with absolute disparity error larger than one in non-occlusion regions are marked red. The percentages of error pixels are listed in Table 3.

Besides, we also compared our results with those produced by other “fused” schemes [20,23,52,53,54,55,56], and the compared results are listed in the Table 3. Our method provides an error rate of 2.61% on the Middlebury datasets, compared to the average error rate 3.27% of the previous state-of-the-art “fused” methods. It is clear that our method performs almost 20% better than other “fused” scheme-based algorithms in the aspect of precision. Furthermore, As shown in Figure 14 and Figure 15, our method achieves comparable results in the following aspects:

- Noise and outliers are significantly reduced, mainly because of the improved luminance consistency term and the texture term.
- The method obtains precise disparities for slanted or highly-curved surfaces of objects with complex geometric characteristics, mainly because of the 3D plane bias term.
- Ambiguous matchings caused by over-segmentation or under-segmentation are overcome and disparity variances become smoother, mainly because of the hybrid smoothness term.

**Table 3:** The percentages of error pixels (absolute disparity error larger than 1 in non-occlusion regions) of our method and other “fused” methods on the Middlebury datasets. “Averages” are the average percentages of error pixels over all images. Compared to the average error rate 3.27% of the previous state-of-the-art “fused” methods, our method provides a lower average error rate of 2.61% on the Middlebury datasets. It performs almost 20% better than other “fused” methods in the aspect of precision.

	The Percentages of Error Pixels (%)									
	Tsukuba	Venus	Teddy	Conse	Wood1	Colth4	Reimdeor	Dools		Averages
**Zhu *et al.*** [20]	1.16	0.14	2.83	3.47	5.38	3.74	5.83	5.46		3.50
**Wang *et al.*** [23]	0.89	0.12	6.39	2.14	4.05	3.81	3.55	2.71		2.96
**Yang *et al.*** [52]	0.94	0.26	5.65	7.18	1.76	2.60	4.43	4.13		3.37
**Jaesik *et al.*** [53,55]	2.38	0.56	5.59	6.28	3.72	2.88	4.04	4.69		3.77
**James *et al.*** [54]	2.90	0.29	2.12	2.83	2.74	2.32	5.02	4.02		2.78
**Ours**	0.79	0.10	3.49	2.06	4.35	1.03	4.31	4.78		2.61

### 4.3. Evaluation Results for Each Term

**Figure 16:**
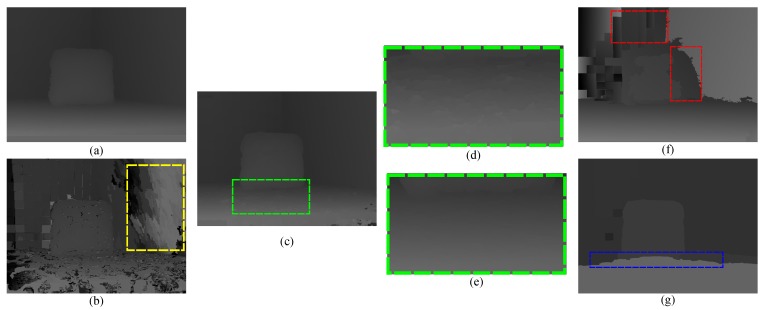
Evaluation results when turning off some terms. (**a**) Our result; (**b**) result without the texture term; (**c**) result without the improved luminance consistency term; (**d**) the detail with an enlarged scale in the green region of (c); (**e**) our result detail with enlarged scale in the same green region of (c); (**f**) result without the hybrid smoothness term; (**g**) result without the 3D plane bias term. Nonocc: non-occlusion regions.

We conducted evaluations to analyze the effect of the individual terms in Equation (4). In each experiment, one term was turned off and the others remained on. First, the texture term was turned off, which meant that the range of the potential disparities for each pixel was no longer restricted by the texture variance and gradient. Ambiguities occurred in textureless and repetitive texture regions without the prior restriction from the data of the depth sensor (see the yellow rectangle in Figure 16b). The average error rate of all images in non-occlusion regions sharply increased to 2.77%. Furthermore, the improved luminance consistency term was turned off by setting $w_{p}^{x}:=0$. Then, this term can be viewed as the conventional one that is easily affected by light variation and causes error matching on the non-Lambertian surface and rich texture regions (see the green rectangle regions in Figure 16c–e). The corresponding average error rate of all images in the non-occlusion regions is 2.37%. Thirdly, the hybrid smoothness term was turned out by replacing by the usual second-order smoothness term [11]. Some artifacts in the red rectangle in Figure 16f were caused by over-segmentation and under-segmentation. Its average error rate sharply increased to 3.02%. Finally, the 3D plane bias term was turned off by setting $\lambda_{p}$:=0. In that case, all 3D object surfaces are assumed as the frontal parallel ones, and the depth map is rather noisy, which makes it difficult to preserve the details at the boundary of objects (see the blue rectangle region in Figure 16g). Its average error rate is 2.14%. The corresponding error statistic analysis on the Middlebury datasets is listed in Table 4. It is clear that our method can obtain the lowest average error rate when all terms turn on (average error rate of 1.61% in non-occlusion regions).

**Table 4:** Error statistic for the Middlebury datasets with different constraint terms turned off. “Averages” are the average percentages of error pixels over all images in different regions.

	Tsukuba				Venus				Teddy				Cones				Averages		
	nonocc	all	disc		nonocc	all	disc		nonocc	all	disc		nonocc	all	disc		nonocc	all	disc
**Texture term off**	2.04	2.12	5.78		0.81	1.03	3.09		5.43	10.2	13.25		2.79	8.26	6.35		2.77	5.40	7.12
**Luminance term off**	1.01	1.65	4.78		0.11	0.25	1.54		5.49	11.20	14.92		2.88	8.47	7.74		2.37	5.39	7.25
**Smoothness term off**	1.39	2.14	4.94		0.85	0.93	2.02		6.57	13.01	14.80		3.28	7.50	7.13		3.02	5.90	7.22
**Plane bias term off**	0.88	1.49	4.86		0.23	0.65	2.27		4.53	9.30	10.63		2.90	7.96	8.96		2.14	4.76	6.68
**All terms on**	0.79	1.21	4.30		0.10	0.21	1.27		3.49	9.04	10.90		2.06	7.05	5.80		1.61	4.37	5.56

### 4.4. Computational Time Analyses

The proposed method was implemented on a PC with Core i5-2500 3.30 GHZ CPU and 4 GB RAM. Table 5 and Table 6 list the running time of the proposed method for all experiments. It is obvious that the computational time is proportional to the image resolution and the scope of potential disparities. For example, it took approximately 1–9 mins to obtain results on Middlebury data and 19–25 mins on the real-world scene datasets. In the future, we aim to implement our method on a GPU to achieve a good balance between accuracy and efficiency.

**Table 5:** Running times for the real-world datasets. The disparity map resolution of all real-world datasets is 1024×960. The corresponding maximum disparity is 107.

	Dragon	Book	Plant	Tableclotd	Board	Box	Kola	Vase	Piggy	Dragon and Piggy
**Running Time (m):**	22.15	20.54	22.16	24.31	21.51	24.26	23.43	22.30	19.44	23.04

**Table 6:** Running times for the Middlebury datasets.

	Tsukuba	Venus	Teddy	Cones	Wood1	Cloth4	Reindeer	Dolls
**Running Time (m):**	1.03	1.19	4.08	4.57	8.18	7.44	7.02	8.31
**Disparity Map Resolution:**	384×288	434×383	450×375	450×375	457×370	433×375	447×370	463×375
**Maximum Disparity:**	15	19	59	59	71	69	67	73

## 5. Conclusions

In this paper, we present an accurate disparity estimation fusion model that “fused” the advantages of the complementary nature of active and passive sensors. Our main contributions are the texture information constraint and the multiscale pseudo two-layer image model. The comparison results show that our method can reduce the error estimate caused by under- or over- segmentation and has good performance in keeping object boundaries compared to using the conventional stereo matching or the depth sensor alone. Furthermore, the proposed method provides an error rate of 2.61% on the Middlebury datasets, compared to the average error rate 3.27% of the previous state-of-the-art “fused” methods. It is clear that our method performs almost 20% better than other “fused” scheme-based algorithms in the aspect of precision. In the future, we will investigate a more accurate method for estimating the disparities of occluded pixels. We also intend to transform our method to a parallel GPU implementation.
